# Manganese Superoxide Dismutase Dysfunction and the Pathogenesis of Kidney Disease

**DOI:** 10.3389/fphys.2020.00755

**Published:** 2020-07-14

**Authors:** Munehiro Kitada, Jing Xu, Yoshio Ogura, Itaru Monno, Daisuke Koya

**Affiliations:** ^1^Department of Diabetology and Endocrinology, Kanazawa Medical University, Uchinada, Japan; ^2^Division of Anticipatory Molecular Food Science and Technology, Medical Research Institute, Kanazawa Medical University, Uchinada, Japan

**Keywords:** manganese superoxide dismutase, acute kidney injury, chronic kidney disease, mitochondria, peroxynitrite, posttranslational modification

## Abstract

The mitochondria are a major source of reactive oxygen species (ROS). Superoxide anion (O_2_^•–^) is produced by the process of oxidative phosphorylation associated with glucose, amino acid, and fatty acid metabolism, resulting in the production of adenosine triphosphate (ATP) in the mitochondria. Excess production of reactive oxidants in the mitochondria, including O_2_^•–^, and its by-product, peroxynitrite (ONOO^–^), which is generated by a reaction between O_2_^•–^ with nitric oxide (NO^•^), alters cellular function via oxidative modification of proteins, lipids, and nucleic acids. Mitochondria maintain an antioxidant enzyme system that eliminates excess ROS; manganese superoxide dismutase (Mn-SOD) is one of the major components of this system, as it catalyzes the first step involved in scavenging ROS. Reduced expression and/or the activity of Mn-SOD results in diminished mitochondrial antioxidant capacity; this can impair the overall health of the cell by altering mitochondrial function and may lead to the development and progression of kidney disease. Targeted therapeutic agents may protect mitochondrial proteins, including Mn-SOD against oxidative stress-induced dysfunction, and this may consequently lead to the protection of renal function. Here, we describe the biological function and regulation of Mn-SOD and review the significance of mitochondrial oxidative stress concerning the pathogenesis of kidney diseases, including chronic kidney disease (CKD) and acute kidney injury (AKI), with a focus on Mn-SOD dysfunction.

## Introduction

The prevalence of chronic kidney disease (CKD) has been increasing worldwide (Eckardt et al., 2013). CKD is characterized by the gradual loss of renal function over a period of months to years, ultimately leading to end-stage renal disease (ESRD). CKD has also been recognized as an independent risk factor for cardiovascular disease (CVD) (Go et al., 2004; Gansevoort et al., 2013). In contrast, acute kidney injury (AKI), also known as acute renal failure, is defined as the sudden and rapid loss of renal function, often within 48 h (KDIGO, 2012). AKI is closely associated with the increased risk of developing CKD, and progression is directly dependent on the severity of AKI (Fiorentino et al., 2018). Therefore, to suppress these outcomes, it will be necessary to have a better understanding of the underlying fundamental mechanisms related to progression and onset of both CKD and AKI.

Reactive oxygen species (ROS) can damage cellular proteins, lipids, and DNA, ultimately leading to cellular dysfunction (Wang et al., 2018). Mitochondria are the major source of endogenous ROS, which depends directly on the metabolic and redox state of the mitochondria. Metabolic state refers to the efficiency of electron transfer from nutrients to molecular oxygen (O_2_). Electrons that are leaked from the respiratory chain react with O_2_ to produce superoxide anions (O_2_^•–^). Mitochondria have ROS scavenging systems via which O_2_^•–^ is converted to H_2_O_2_ by the actions of superoxide dismutase (SOD), including Cu/Zn-SOD and manganese superoxide dismutase (Mn-SOD) (Mailloux, 2015; Kim et al., 2017; Wang et al., 2018). Further decomposition of H_2_O_2_ to H_2_O and O_2_ is catalyzed by other antioxidative enzymes within the mitochondria, including glutathione peroxidase (GPx) and peroxiredoxin (PRx)/thioredoxin (TRx) (Mailloux, 2015; Kim et al., 2017; Wang et al., 2018). At low levels, ROS are second messengers for signal transduction and may serve to regulate cellular adaptation against several stressors. However, oxidative stress results when ROS production exceeds homeostatic levels due to an imbalance between ROS production and decomposition.

The kidney is a highly metabolic organ with high levels of oxidation within cellular mitochondria. Renal tissues have a large energy demand, most notably within the proximal tubular cells; this is related to their role in reabsorbing critical nutrients after glomerular filtration. As such, the kidney is particularly vulnerable to damage caused by oxidative stress. Previous reports have shown that mitochondrial oxidative stress may be directly linked to mechanisms underlying the onset and progression of both CKD and AKI (Che et al., 2014; He et al., 2017). Excess mitochondrial ROS may promote mitochondrial dysfunction, ATP depletion, inflammation, and apoptosis in association with kidney disease (Che et al., 2014; He et al., 2017). Among ROS scavenging enzymes in the mitochondrial matrix, Mn-SOD has a primary responsibility for O_2_^•–^ scavenging within mitochondria; as such, dysfunction of Mn-SOD results in mitochondrial oxidative stress. Collective experimental evidence has suggested a relationship between Mn-SOD dysfunction and the pathogenesis of kidney disease. In this review, we consider the significance of mitochondrial oxidative stress on the pathogenesis of kidney disease, including CKD and AKI, with a focus on the role of Mn-SOD dysfunction.

## Biological Function of Manganese Superoxide Dismutase on Reactions and Transformations of Superoxide Anion

**FIGURE 1:**
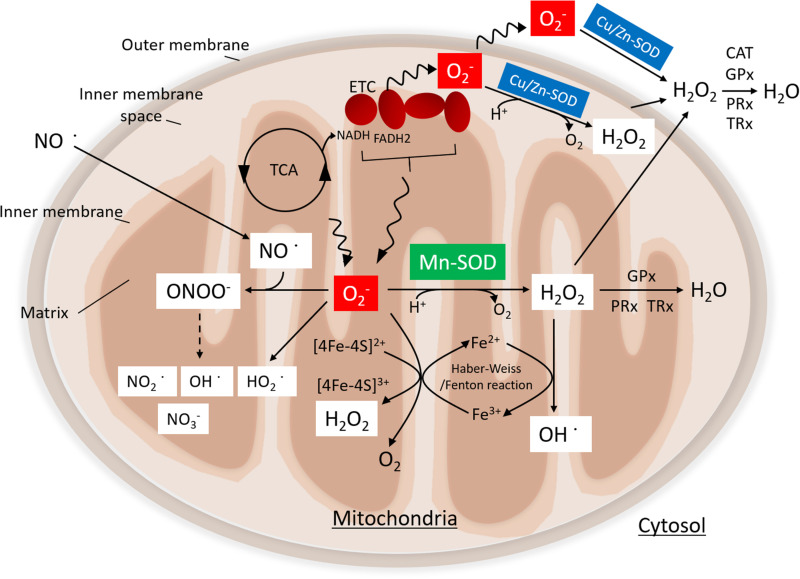
Function of manganese superoxide dismutase (Mn-SOD). Superoxide (O_2_^•–^) is produced by the electron transport chain (ETC) during nutrient metabolism by the tricarboxylic acid (TCA) cycle. Mn-SOD is localized in the mitochondrial matrix, where it catalyzes the dismutation of O_2_^•–^ to H_2_O_2_. By contrast, Cu/Zn-SOD, which is located in the inner membrane space of mitochondria, catalyzes the conversion of O_2_^•–^ to hydrogen peroxide (H_2_O_2_). H_2_O_2_ is further metabolized by the glutathione peroxidase (GPx) and the peroxiredoxin (PRx)/thioredoxin (TRx) system in mitochondrial matrix or by catalase (CAT) GPx and PRx/TRx in the cytosol. O_2_^•–^ and nitric oxide (NO^•^) can react to form peroxynitrite (ONOO^–^), giving rise to nitrogen dioxide (NO_2_^•^) and hydroxyl radical (OH^•^); eventually, stable nitrite (NO_3_^–^) is produced. H_2_O_2_ is converted to OH via the Fenton/Haber–Weiss reaction. O_2_^•–^ can reduce ferric iron (Fe^3+^) to ferrous iron (Fe^2+^) in iron–sulfur centers of proteins, ultimately resulting in the production of H_2_O_2_. Moreover, the protonation of O_2_^•–^ generates the hydroperoxyl radical, HO_2_.

Human Mn-SOD, encoded by the *sod2* gene, is located on chromosome 6q25.3 (Creagan et al., 1973) (Figure 1). The sequence of Mn-SOD is highly conserved, with over 40% homology among proteins from human, yeast, and *Escherichia coli* (Barra et al., 1984). Mn-SOD is a tetrameric enzyme with four identical subunits, each harboring a manganese ion (Mn^2+^/Mn^3+^) as a cofactor. Mn-SOD is located primarily within the mitochondrial matrix (Borgstahl et al., 1992; Karnati et al., 2013). By contrast, Cu/Zn-SOD is localized to the mitochondrial inner membrane space.

O_2_^•–^ production in mitochondria is closely linked to mitochondrial production of adenosine triphosphate (ATP), which takes place via electron transfer linked to nutrient (glucose, amino acids, and fatty acids) metabolism. Electrons are by-products of the tricarboxylic acid (TCA) cycle enzymes and respiratory complexes that promote a univalent reduction of oxygen (O_2_) to O_2_^•–^. Superoxide anion (O_2_^•–^) is an important signaling molecule but one that can be toxic at high concentrations. SOD enzymes, including Mn-SOD and Cu/Zn-SOD, catalyze the dismutation of O_2_^•–^ to H_2_O_2_ in the mitochondrial matrix and the intermembrane space, respectively (Kawamata and Manfredi, 2010; Karnati et al., 2013; Mailloux, 2015). The GPx and PRx/TRx systems convert H_2_O_2_ to H_2_O within the mitochondrial matrix. Alternatively, H_2_O_2_ that has diffused into the cytoplasm is metabolized by catalase, GPx, and PRx/TRx. O_2_^•–^ can also react with nitric oxide (NO^•^) to produce peroxynitrite (ONOO^–^). The decomposition of ONOO^–^ gives rise to highly oxidizing intermediates, including nitrogen dioxide (NO_2_^•^), and hydroxyl radical (OH^•^); eventually, stable nitrite (NO_3_^–^) is produced (Tharmalingam et al., 2017; Radi, 2018). As such, elevated levels of O_2_^•–^ may result in decreased bioavailability of NO and an increased toxic ONOO^–^ production. Additionally, O_2_^•–^ may reduce ferric iron (Fe^3+^) to ferrous iron (Fe^2+^) in iron–sulfur centers in critical proteins, which may lead to enzyme inactivation and concomitant loss of Fe^2+^ from the enzymes, thereby promoting production of H_2_O_2_ (Imlay, 2003). H_2_O_2_ may then react with Fe^2+^ to produce hydroxy radicals (OH^•^) via the Fenton/Haber–Weiss reaction. Moreover, protonation of O_2_^•–^ may promote formation of the reactive hydroperoxyl radical HO_2_.

Oxidative stress induced by the imbalance between ROS production and the scavenging capacity of antioxidant protection mechanisms in the mitochondria leads to the inactivation of endogenous antioxidant systems, impairment of the electron transport, uncoupling of oxidative phosphorylation, and altered membrane permeability. As such, internal oxidative stress-mediated damage may be the main cause of mitochondrial dysfunction. Mn-SOD is the primary antioxidative enzyme that is responsible for scavenging O_2_^•–^ in the mitochondrial matrix. Therefore, Mn-SOD dysfunction may result in overproduction of highly reactive oxidants, such as ONOO^–^ and OH, which may result in mitochondrial dysfunction and disease development.

## Transcriptional Regulation of Manganese Superoxide Dismutase

The *sod2* gene has three major regulatory regions, namely, a proximal promoter, a 5′ upstream enhancer region, and an intronic enhancer region, which promotes activation or repression of *sod2* gene expression (Xu et al., 2002).

### Proximal Promotor of the *sod2* Gene

The basic proximal promoter of the *sod2* gene has no classical TATA or CAAT box, although it is enriched in CG repeats that contain binding sites for specificity protein 1 (Sp1) and activator protein 2 (AP-2) (Zhu et al., 2001; Xu et al., 2002). Sp1 binding to the promoter activates *sod2* gene transcription, whereas AP-2 suppresses the transcriptional activity via competition with Sp1 at its binding site or by limiting its bioavailability via AP-2/Sp1 complex formation. As such, Sp1 and AP-2 regulate the basal level of *sod2* gene transcription via antagonistic mechanisms at the proximal promoter region.

### 5′ Upstream Enhancer Region of the *sod2* Gene

The 5′ upstream enhancer region of the *sod2* gene includes binding sites for activating protein 1 (AP-1) (Borrello and Demple, 1997), cyclic AMP response element-binding protein (CREB) (Kim et al., 1999), nuclear factor, erythroid 2-like 2 (Nrf2) (Taylor et al., 2008), forkhead box O3 (FoxO3a) (Kops et al., 2002), nuclear factor-kappa B (NF-κB) (Warner et al., 1996), and hypoxia-inducible factor 1α (HIF-1α) (Gao et al., 2013). Binding of transcription factors at these sites within the *sod2* 5′ enhancer leads to an interaction with the basal transcriptional machinery of the proximal promoter, including Sp1. Nrf2 binds to the antioxidant responsive element (ARE) and acts as a master regulator of ARE-responsive antioxidant genes, including *sod2* at homeostasis and in response to induction (Taylor et al., 2008; Li et al., 2015). FoxO-binding elements were identified upstream of the *sod2* transcriptional start site; however, only the site at position −1,249 mediates FoxO3a-dependent transcription of *sod2* gene in quiescent cells (Kops et al., 2002). In pathological conditions, activation of Akt (protein kinase B) promotes nuclear exclusion of FoxO3a via phosphorylation, thereby downregulating the transcription of *sod2* (Kato et al., 2006; Li et al., 2006). FoxO3 is deacetylated by sirtuins, including SIRT1, SIRT2, and SIRT3, resulting in enhanced DNA binding and *sod2* gene transcription (Jacobs et al., 2008). HIF-1α represses *sod2* gene transcription in association with renal clear cell carcinoma (Gao et al., 2013). High levels of mitochondrial O_2_^•–^ that result from the repression of *sod2* gene expression lead to HIF-1α stabilization. Moreover, peroxisome proliferator-activated receptor-γ coactivator-1α (PGC-1α) also displays antioxidant defense responses via its role in promoting upregulated expression of antioxidative enzymes, including *sod2* (Valle et al., 2005; St-Pierre et al., 2006; Lu et al., 2010). PGC-1α also plays an important role in mitochondrial biogenesis and respiration (Wu et al., 1999; Lin et al., 2005). Ding et al. (2017) reported that knockdown of SIRT3 inhibited osteogenic differentiation via its role in decreasing the stability of PGC-1α by increasing its acetylation and via downregulated expression of Mn-SOD; this results in elevated levels of ROS and reduced mitochondrial biogenesis and function. Cherry et al. (2014) reported that PGC-1α promotes mitochondrial biogenesis and induces expression of antioxidative enzyme, most notably Nrf2-mediated Mn-SOD expression in the liver of mice of *Staphylococcus aureus*-induced peritonitis.

Binding sites for p53 were identified in the 5′ upstream region of the *sod2* gene (Pani et al., 2000; Drane et al., 2001). p53 exerts its inhibitory action via the prevention of Sp1 binding to the proximal promoter of *sod2* gene (Dhar et al., 2006). Additionally, high levels of p53 expression serve to suppress *sod2* expression, whereas low levels of p53 expression promote NF-κB binding to the enhancer region of the *sod2* gene and increasing *sod2* transcription (Dhar et al., 2010). In contrast, Hussain et al. (2004) reported that the promoter of *sod2* contains p53 consensus binding sites, which located at −2,009 to −2,032 bp upstream of the transcription start site and induction of p53 leads to increased promoter activity and gene expression.

### Intronic Enhancer Region of the *sod2* Gene

The intronic enhancer contains a primary binding site for NF-κB (Jones et al., 1997; Xu et al., 1999). NF-κB positively regulates transcription via binding to the intronic enhancer region, which is located in the second intron of the *sod2* gene by promoting interactions with CCAAT/enhancer-binding protein beta (C/EBP-β; Xu et al., 1999; Jones et al., 1997). NF-κB binding at the intronic enhancer region also enhances transcriptional activity of Sp1 in the proximal promoter of *sod2* (Ennen et al., 2011).

## Posttranslational Regulation of Manganese Superoxide Dismutase Activity

In addition to transcriptional regulation, previous reports indicated that Mn-SOD function is regulated by posttranslational modifications, including nitration, acetylation, phosphorylation, and glutathionylation.

### Nitration

**FIGURE 2:**
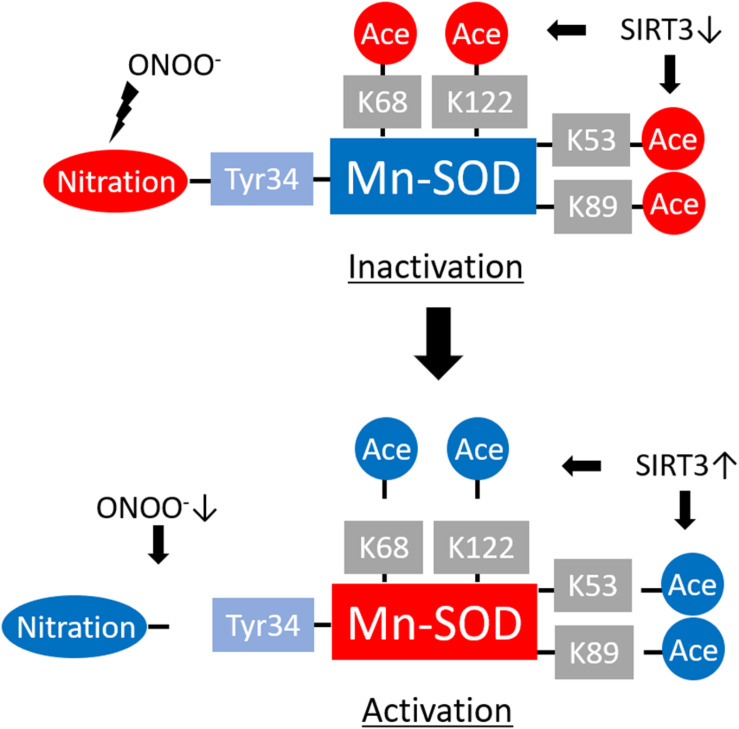
Regulation of manganese superoxide dismutase (Mn-SOD) enzyme activity by translational modifications. ONOO^–^ promotes Mn-SOD inactivation by nitrating the critical Tyr34 at the enzyme active site. Acetylation of Mn-SOD at K68, K122, K53, and K89 residues also induces Mn-SOD inactivation. Reduced levels of ONOO^–^ and activation of NAD-dependent deacetylase sirtuin-3 (SIRT3) lead to Mn-SOD reactivation through denitrification or deacetylation of the target residues.

NO, a ubiquitous intracellular messenger, plays crucial roles in a variety of biological processes (Beckman and Koppenol, 1996). Overproduction of NO and O_2_^•–^ leads to the formation of the extremely reactive ONOO^–^, which can result in lipid peroxidation, DNA damage, and protein nitration. ONOO^–^ can generate covalent modifications of tyrosine (Tyr) residues in proteins, thereby reducing their functionality (Figure 2).

ONOO^–^ leads to Mn-SOD deactivation via nitration of the critical active-site Tyr34 residue (MacMillan-Crow et al., 1998; Yamakura et al., 1998; Yamakura and Kawasaki, 2010) (Figure 2). Mn-SOD protects cells by O_2_^•–^ scavenging, thereby preventing the interaction of O_2_^•–^ with NO. However, Mn-SOD deactivation leads to the amplification of oxidative stress via accumulation of more O_2_^•–^ that promotes not just only ONOO^–^ formation in the mitochondria but also both the onset and progression of several diseases.

### Acetylation

Nε-lysine acetylation is recognized as a critical posttranslational modification that regulates protein function, most notably that of histones (Narita et al., 2019). Non-histone protein acetylation modulates numerous cellular processes, including gene transcription, DNA damage and repair, cell division, signal transduction, protein folding, autophagy, and metabolism.

Sirtuins (SIRTs), which are NAD-dependent protein deacetylases, play significant roles in the regulation of protein acetylation (Guarente, 2011). SIRT3, SIRT4, and SIRT5 have been identified in the mitochondria (Guarente, 2011; Kitada et al., 2019). The deacetylase activity of SIRT3 is substantially higher than that of SIRT4 or SIRT5; as such, SIRT3 plays a critical role in mitochondrial biology and pathophysiological processes, including redox status, via its capacity to regulate acetylation of lysine (K) residues in mitochondrial proteins (Guarente, 2011; van de Ven et al., 2017; Kitada et al., 2019). Mn-SOD is among the target proteins of SIRT3. Chen et al. (2011) found that Mn-SOD K68 is an important site for SIRT3 regulation in human cells, whereas Tao et al. (2010) identified K122 and Qiu et al. (2010) identified K53 and K89 in Mn-SOD as the main sites of Sirt3 deacetylation in mouse cells (Figure 2). Overall, we conclude that SIRT3 deacetylates Mn-SOD at several lysine residues. SIRT3-mediated deacetylation results in enhanced Mn-SOD activity within a given nutrient and redox state and under various pathophysiological conditions. This results in decreased mitochondrial oxidative stress and prevents the progression of cellular aging and aging-related disease.

### Other Modifications

Mn-SOD can also be phosphorylated, and this process has a direct impact on enzyme function. Candas et al. (2013) reported that the CyclinB1/CDK1-induced phosphorylation of Mn-SOD at Ser106 led to Mn-SOD activation and increased stability, which was associated with improved mitochondrial function and cellular resistance to apoptosis due to radiation. Additionally, cyclin-dependent kinase 4 (CDK4) located in mitochondria directly phosphorylates Mn-SOD at Ser106, resulting in increased Mn-SOD activity and mitochondrial respiration, notably in skin tissues of mice receiving whole-body low-dose ionizing radiation (Jin et al., 2015). The phosphorylation at Ser106 of Mn-SOD enhances its tetrameric conformation, stability, and enzymatic activity. By contrast, Hopper et al. (2006) reported an inverse correlation between phosphorylation and activity of Mn-SOD in mitochondria isolated from pig heart, and Ca^2+^-induced dephosphorylation increased Mn-SOD enzymatic activity. Thus, further studies are necessary to clarify how the phosphorylation or dephosphorylation of Mn-SOD is involved in the regulation of Mn-SOD activity.

Additionally, Zhao et al. (2017) identified Mn-SOD oxidation at histidine (His54 and His55), tyrosine (Tyr58), tryptophan (Trp147, Trp149, Trp205, and Trp210), and asparagine (Asn206 and Asn209) residues in human kidney tissues through mass spectrometry. In renal carcinoma cells, Mn-SOD oxidation at histidine (His54 and His55), tyrosine (Tyr58), and tryptophan (Trp147 and Trp149) residues was enhanced and associated with Mn-SOD deactivation (Zhao et al., 2017). John et al. (2009) also found that oxidation at His54 and His55 was associated with Mn-SOD deactivation in human medulloblastoma cells (John et al., 2009).

S-glutathionylation is the term that describes the addition of glutathione to cysteine residues of proteins; this process prevents irreversible oxidation of protein thiols and regulates a diverse array of cellular processes (Mailloux and Willmore, 2014). Mn-SOD can undergo S-glutathionylation within the mitochondria. Hopper et al. (2006) reported S-glutathionylation of recombinant rat Mn-SOD that was generated in *E. coli*. Mn-SOD cysteine (Cys) 196 was identified as a target of S-glutathionylation in the rat (Castellano et al., 2009). Patil et al. (2013) demonstrated that diamide, a biochemical that oxidizes glutathione and thus promotes protein glutathionylation, promoted reversible reductions in Mn-SOD activity when added to cultured rat renal tubular cells or when used to perfuse rat kidney *in vivo*.

## Physiological Significance of Manganese Superoxide Dismutase on Kidney Disease in Animal Studies

### Lessons From Manganese Superoxide Dismutase Gene-Altered Mice

Studies featuring knockout (KO) of Mn-SOD activity via inactivating mutations or due to the complete elimination of Mn-SOD expression have been performed in mice. These manipulations have resulted in massive oxidative stress and neonatal death associated with cardiomyopathy, neurodegeneration, lipid accumulation in the liver and skeletal muscle, and metabolic acidosis (Li et al., 1995; Holley et al., 2012). There were no obvious alterations in mitochondrial structure in Mn-SOD gene-deleted (^–/–^) mice; however, this gene deletion had a substantial impact on mitochondrial enzyme activity, including significant reductions in the activity of both succinate dehydrogenase and aconitase when compared with wild-type mice. By contrast, Lebovitz et al. (1996) previously generated a line of Mn-SOD knockout mice (SOD2^mlBCM^/SOD2^mlBCM^), in which the phenotype of mice is different from Mn-SOD knockout mice produced by other groups. Their mice survived longer than other group’s mice and exhibited extensively swollen and damaged mitochondria within degenerating neurons and cardiac myocytes. These discrepancies on the phenotype of Mn-SOD knockout mice possibly depend on differences in the molecular constructs used to generate the deletions and in the genetic backgrounds on which the mutations were being bred. Williams et al. (1998) reported that Mn-SOD heterozygotes (^±^) exhibited a 50% decrease in Mn-SOD activity, which was associated with impaired function and increased oxidative stress in the mitochondria in liver cells. Van Remmen et al. (2003) also reported increased and lifelong oxidative damage to DNA in liver, brain, and heart tissues of the Mn-SOD^±^ mice; oxidative damage to DNA was associated with an increased incidence of cancer in these mice.

The physiological and pathological roles of Mn-SOD concerning renal function have been investigated using the heterozygous Mn-SOD^±^ mice and with renal-specific Mn-SOD KO mice. Rodriguez-Iturbe et al. (2007) demonstrated oxidative stress and renal interstitial inflammation in a cohort of Mn-SOD^±^ mice. These alterations were found to be compatible with accelerated renal senescence and the development of salt-sensitive hypertension. Additionally, Parajuli et al. (2011) created kidney-specific Mn-SOD KO mice from two different transgenic mouse lines. Specifically, “floxed” Mn-SOD mice (exon 3 of Mn-SOD allele flanked with LoxP sites in introns 2 and 3) were crossed with transgenic Ksp1.3/Cre (kidney Cre) mice. The Ksp1.3/Cre transgenic mice expressed Cre recombinase specifically in distal tubules, collecting ducts, loops of Henle, ureteric buds, and the developing genitourinary tract of the kidney. As such, the impact of the Mn-SOD KO is limited to these specific parts of the kidney. Kidney-specific Mn-SOD KO mice exhibited normal renal function; however, the localized Mn-SOD KO resulted in modest renal damage, including tubular dilation, epithelial cell enlargement, the formation of casts within the tubular lumen, and the accumulation of nitrotyrosine-modified protein. These data indicate that Mn-SOD plays a crucial role in maintaining redox homeostasis in kidney mitochondria.

### Kidney Disease Animal Model Studies

**FIGURE 3:**
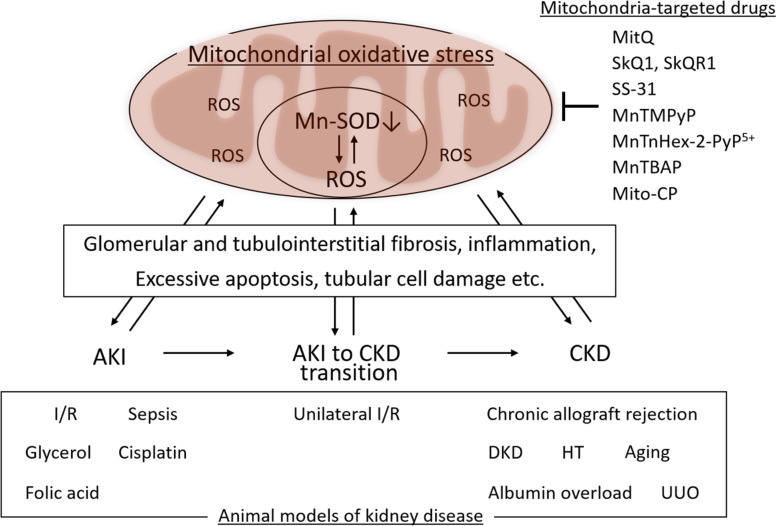
Mitochondrial oxidative, manganese superoxide dismutase (Mn-SOD) dysfunction, and kidney disease. Mitochondrial oxidative stress associated with Mn-SOD dysfunction in the kidney is one of the key factors underlying the pathogenesis of kidney disease, including acute kidney injury (AKI), chronic kidney disease (CKD), as well as the AKI to CKD transition through glomerular and tubulointerstitial fibrosis, inflammation, excessive apoptosis of renal cells, and tubular cell damage. Several mitochondria-targeted drugs may improve the kidney disease through the improvement of mitochondrial oxidative stress. I/R, Ischemia/reperfusion; DKD, diabetic kidney disease; HT, hypertension; UUO, unilateral ureteral obstruction.

Mitochondrial oxidative stress associated with Mn-SOD dysfunction in the kidney is involved in the pathogenesis of several kidney diseases, including AKI and CKD, as well as in the AKI to CKD transition. Mn-SOD dysfunction has been associated with glomerular and tubulointerstitial fibrosis, inflammation, excess apoptosis of renal cells, and tubular cell damage, as one of the sources of ROS in the mitochondria (Figure 3). We focused on the change of Mn-SOD in the kidney, such as posttranslational modification, activity, and expression levels, in animal models of kidney disease, and showed the summary in Table 1.

**TABLE 1:** Change of renal manganese superoxide dismutase (Mn-SOD) in animal models of kidney disease.

Type of kidney disease	Animal models of kidney disease	Change of Mn-SOD in the kidney	References
AKI	**I/R-induced AKI models**		
	I/R mice	Tyrosine nitration of Mn-SOD ↑	Cruthirds et al., 2003
		Acetylated Mn-SOD ↑	Ouyang et al., 2019
	**Sepsis-associated AKI models**		
	CLP mice	Mn-SOD activity ↓	Patil et al., 2014
		Mn-SOD expression/activity ↓	Chen G. D. et al., 2018
	CLP rats	Acetylated Mn-SOD ↑	Xu, 2016
	**Other AKI models**		
	Glycerol-induced AKI rats	Mn-SOD expression ↓	Homsi et al., 2011
	Cisplatin-induced AKI mice	Mn-SOD expression ↓	Rattanavich et al., 2013
	Folic acid-induced AKI mice	Mn-SOD expression ↓	Zhang et al., 2019
AKI to CKD transition	Unilateral I/R mice	Mn-SOD expression ↓	Zhang et al., 2018
CKD	**Chronic allograft rejection and associated nephropathy models**		
	Chronic allograft rejection of rats	Tyrosine nitration of Mn-SOD ↑	MacMillan-Crow et al., 1996, 2001
	**DKD models**		
	db/db mice	Tyrosine nitration of Mn-SOD ↑	Kitada et al., 2011
		Mn-SOD expression ↓	Lu et al., 2013; Hou et al., 2018
	STZ-induced diabetic Apo E^–/–^ mice	Tyrosine nitration of Mn-SOD ↑	Xu et al., 2006
		Mn-SOD expression ↓	Yi et al., 2011
	Zucker diabetic fatty rats	Acetylated Mn-SOD ↑	Ogura et al., 2018
	STZ-induced diabetic mice	Mn-SOD expression ↓	Spencer et al., 2018; Li et al., 2019
	A high-sugar/high-fat or cholesterol diet with STZ-induced diabetic rats	Mn-SOD expression ↓	Onozato et al., 2004; Chen P. et al., 2018; Zayed et al., 2018
	BTBR ob/ob mice	Mn-SOD expression ↓	Morigi et al., 2020
	Diabetic SHRs	Mn-SOD expression ↓	Tang et al., 1997
	KK/Ta-Akita mice	Mn-SOD expression ↑	Fujita et al., 2009
	**Hypertension-related kidney injury models**		
	Angiotensin II-induced hypertension rats	Tyrosine nitration of Mn-SOD ↑	Guo et al., 2003
	SHRs treated with the NOS inhibitor	Mn-SOD expression ↑, Mn-SOD activity ↑	Majzunova et al., 2019
	SHRs fed high-fat diet	Mn-SOD expression ↓	Chung et al., 2012
	**Aging-related kidney injury models**		
	24 months old mice (vs. 12 months old mice)	Mn-SOD expression ↓	Lim et al., 2012
	Klotho^–/–^ mice	Mn-SOD expression ↓	Kimura et al., 2018
	16 months old mice (vs. 8 weeks old mice)	Mn-SOD expression ↓	Hou et al., 2016
	20 months old mice (vs. 3 months old mice)	Mn-SOD expression ↓	Teissier et al., 2019
	52 weeks old rats (vs. 13 weeks old rats)	Mn-SOD expression ↑	Gomes et al., 2009
	**Other CKD models**		
	Albumin-overload mice	Mn-SOD expression ↓	Zhuang et al., 2015; Jia et al., 2016
	UUO mice	Mn-SOD expression ↓	Li et al., 2017; Liu et al., 2017

*AKI, Acute kidney disease; CKD, chronic kidney disease; I/R, ischemic/reperfusion; CLP, cecal ligation and puncture; STZ, streptozotocin; SHRs, spontaneously hypertensive rats; UUO, unilateral ureteral obstruction.*

#### Ischemia/Reperfusion-Induced Acute Kidney Injury

Increased oxidative stress is a well-known factor that contributes to renal ischemia/reperfusion (I/R) injury (Noiri et al., 2001). Mitochondria are remarkably sensitive to I/R injury, as I/R rapidly promotes the generation of O_2_^•–^ and other ROS, including ONOO^–^, within the injured mitochondria (Walker et al., 2000).

In the kidney of a rodent model of I/R AKI, Cruthirds et al. (2003) reported an acute increase in oxidative stress associated with increased levels of mitochondrial protein nitration, including Mn-SOD and cytochrome c, which resulted in Mn-SOD deactivation and mitochondrial dysfunction. Additionally, renal SIRT3 activity was reduced in response to I/R-induced AKI compared with activity observed among those in the control/sham-operated group. This was also accompanied by increased acetylation of both Mn-SOD and p53 (Ouyang et al., 2019). Taken together, these results suggest that inactivation of Mn-SOD induced by posttranslational modifications, such as nitration and acetylation, results in accelerated renal mitochondrial oxidative stress and contributes to the progression of I/R-induced AKI.

Le Clef et al. (2016) established a unilateral renal I/R model to study the associated mechanisms and therapies directed toward inhibiting the AKI to CKD transition in mice. Zhang et al. (2018) demonstrated that the mitochondrial complex-1 inhibitor, rotenone, slows the progression of AKI to CKD. By the mitochondrial complex-1 inhibitor, the expression of Mn-SOD and ATP synthase subunit β, mitochondrial DNA copy number, were restored, and inflammation and fibrosis were suppressed, in this model. These data suggest that mitochondrial O_2_^•–^ may play a crucial role in promoting the pathogenesis associated with the transition from AKI to CKD.

#### Sepsis-Associated Acute Kidney Injury

Oxidative stress plays a crucial role in the development of mitochondrial dysfunction in response to septic AKI (Schrier and Wang, 2004). Patil et al. (2014) reported a decrease in renal Mn-SOD activity in association with the experiment in sepsis mice, which led to mitochondrial oxidative stress. Treatment with Mito-TEMPO, a mitochondrial-targeted mimetic of SOD, resulted in improvements in sepsis-induced AKI, which were directly associated with increased activation of Mn-SOD and decreased mitochondrial oxidative stress. Additionally, Chen G. D. et al., 2018 demonstrated that renal Mn-SOD expression and activity were reduced in sepsis-induced AKI and that insulin therapy reversed mitochondrial dysfunction via the suppression of the oxidative stress associated with upregulation of Mn-SOD. Furthermore, Xu (2016) found that SIRT1/SIRT3 activity was reduced in the renal tubular epithelial cells of rats with sepsis-associated AKI. This finding was accompanied by increasing levels of acetylated Mn-SOD, swollen mitochondria, and elevated levels of cellular apoptosis. Interestingly, activation of SIRT1 via treatment with resveratrol partially restored SIRT3 activity, which led to improved mitochondrial function and reduced levels of apoptosis. Taken together, these data indicate that Mn-SOD dysfunction and associated mitochondrial oxidative stress are both closely linked to mechanisms promoting disease pathogenesis in sepsis-related AKI.

#### Other Acute Kidney Injury Models

Glycerol-induced AKI results in increased expression of phosphorylated p53 in renal tissues in association with elevated levels of tubular cell apoptosis and oxidative stress due to decreased expression of antioxidative enzymes, including Mn-SOD (Homsi et al., 2011). Treatment with the p53 inhibitor, pifithrin-α, reversed features of glycerol-induced renal injury, including tubular cell apoptosis and oxidative stress via its capacity to restore Mn-SOD expression in the kidney (Homsi et al., 2011).

Oxidative stress and mitochondrial dysfunction related to ATP depletion have been observed in response to cisplatin-induced cellular injury in the kidney (Arany and Safirstein, 2003). Rattanavich et al. (2013) reported that cisplatin treatment results in diminished expression of renal Mn-SOD via the enhanced expression of phospho-p66ShcA and phospho-Foxo3A in studies featuring heterozygous p66ShcA (^±^) mice. Specifically, increased expression of phospho-p66ShcA and phospho-Foxo3A was detected in the renal tissue of wild-type mice treated with cisplatin in association with reduced expression of both Mn-SOD and catalase. By contrast, renal tissue from cisplatin-treated p66ShcA^±^ mice revealed attenuated phosphorylation of p66ShcA and Foxo3a, together with a restored expression of Mn-SOD and catalase.

In the folic acid (FA)-induced AKI model, mitochondrial DNA copy number and expression of mitochondrial cytochrome c oxidase subunit 1, mitochondrial NADH dehydrogenase subunit 1, and Mn-SOD in the kidney were all reduced. These findings implicate mitochondrial dysfunction and oxidative stress in the pathogenesis of FA-induced AKI (Zhang et al., 2019). However, pretreatment with rotenone aggravated the renal injury, mitochondrial damage, and oxidative stress in this mouse model of AKI.

#### Chronic Renal Allograft Rejection

Chronic allograft rejection is currently the most important cause of renal transplant failure (Shrestha and Haylor, 2016). At the tissue level, increased oxidative stress observed in response to I/R may contribute to chronic allograft rejection and associated nephropathy. MacMillan-Crow et al. (1996, 2001) previously reported that endogenous tyrosine nitration and inactivation of Mn-SOD were observed in both human recipients and rat models of chronic renal allograft rejection. These data suggest that deactivation of Mn-SOD by ONOO^–^ results in a progressive increase in the production of ONOO^–^, leading to irreversible oxidative injury to mitochondria in the renal allograft.

#### Diabetic Kidney Disease

Diabetic kidney disease (DKD) is among the main causes of ESRD and an independent risk factor for CVD (Ninomiya et al., 2009). Mitochondrial dysfunction has been linked to the pathogenesis of DKD, and mitochondrial oxidative stress is closely associated with mitochondrial dysfunction (Sharma, 2017; Xu et al., 2020). Dysfunction or decreased expression of Mn-SOD is one of the major factors associated with mitochondrial oxidative stress in diabetic kidney and, as such, with the pathogenesis of DKD.

The increased detection of nitrotyrosine in proximal tubules in renal biopsy samples from diabetic patients compared with non-diabetic control suggested that oxidant injury within the proximal tubules may play a critical role in the pathogenesis of DKD (Thuraisingham et al., 2000). Reduction of Mn-SOD activity in the kidney due to posttranslational modifications and oxidative stress has also been shown in several diabetic animal models. We previously demonstrated that mitochondrial oxidative stress is enhanced in the kidney of diabetic db/db mice when compared with kidneys from non-diabetic db/m controls. These observations were linked to tyrosine nitration of Mn-SOD and the associated reduction in enzyme activity (Kitada et al., 2011). Administration of resveratrol reversed the level of mitochondrial oxidative stress by limiting the extent of nitration, thereby increasing Mn-SOD activity in the diabetic kidney (Kitada et al., 2011). Similarly, in the kidneys of STZ-induced diabetic apolipoprotein E^–/–^ mice, a decrease in Mn-SOD activity was associated with an increase in Mn-SOD Tyr-34 nitration (Xu et al., 2006). Antagonists of thromboxane A_2_ receptors limited diabetes-induced renal injury, which was also accompanied by a reduction of tyrosine nitration (Xu et al., 2006). Additionally, we found that mitochondrial oxidative stress was induced by a reduction in Mn-SOD activity via increased acetylation at K68; this finding was associated with a decreased intracellular NAD^+^/NADH ratio and decreased SIRT3 activity accompanied by increased expression of CD38, a NAD-degrading enzyme, in the kidneys of Zucker diabetic fatty rats (Ogura et al., 2018). Additionally, administration of the CD38 inhibitor, apigenin, resulted in improved mitochondrial oxidative stress through SIRT3 activation and Mn-SOD deacetylation (Ogura et al., 2020).

The expression of Mn-SOD is reduced in several diabetic animal models, including STZ-induced type 1 diabetes (Spencer et al., 2018; Li et al., 2019), a high-sugar/high-fat or cholesterol diet with STZ-induced diabetes (Onozato et al., 2004; Chen P. et al., 2018; Zayed et al., 2018), Apo E^–/–^ mice with STZ-induced diabetes (Yi et al., 2011), db/db mice (Lu et al., 2013; Hou et al., 2018), BTBR ob/ob mice (Morigi et al., 2020), and diabetic spontaneously hypertensive rats (SHR) (Tang et al., 1997). This has been associated with increased oxidative stress in kidney mitochondria. However, the diabetic KK/Ta-Akita mice feature the downregulation of cytosolic Cu/Zn-SOD and extracellular Cu/Zn-SOD, but not mitochondrial Mn-SOD, in the kidney (Fujita et al., 2009). Additionally, Dugan et al. (2013) demonstrated no increased renal disease in STZ-induced diabetes in heterozygous Mn-SOD^±^ mice over that observed in STZ-induced WT mice.

#### Hypertension-Related Kidney Injury

Oxidative stress in blood vessels and the kidney due to chronic hypertension may result from the dysregulated responses of several vasoconstrictor mechanisms, including those associated with angiotensin II stimulation or with dysfunction of nitric oxide synthase (eNOS). Angiotensin II-induced hypertension promotes an increase in O_2_^•–^ generation via the activation of NADPH oxidase (Wang et al., 2001; Onozato et al., 2002). The antioxidant response is impaired in hypertensive animals, which results in oxidative stress followed by the development of end-organ damage, including ESRD. Guo et al. (2003) demonstrated that angiotensin II-induced hypertension in rats was accompanied by increased tyrosine nitration and thus deactivation of Mn-SOD that serves to augment oxidative stress in the kidney. Activation of NADPH oxidase also induces the production of O_2_^•–^, and ONOO^–^ also may induce eNOS uncoupling, leading to overproduction of O_2_^•–^ as opposed to NO.

Protein nitration was increased in the renal cortex of SHRs treated with the NOS inhibitor, L-NAME (Majzunova et al., 2019). Renal expression of Mn-SOD was increased in SHR in response to treatment with L-NAME, although total SOD activity remained unchanged (Majzunova et al., 2019). These data suggest that the impaired antioxidant response combined with a deficiency in NO may promote oxidative damage of proteins in the kidney via the production of ONOO^–^ in SHRs. By contrast, SHRs that fed a high-fat diet (HFD) developed renal lipotoxicity, insulin resistance, and hypertension in association with a decrease in PPARα expression. This leads to Akt activation, increased FoxO3a phosphorylation, suppression of the PGC-1α-ERR-1α pathway, reduced expression of Mn-SOD, and increased oxidative stresses (Chung et al., 2012).

#### Aging-Related Kidney Dysfunction

Aging-related changes lead to the functional decline of several organs, including the kidney. Oxidative stress, particularly mitochondrial oxidative stress, has been recognized as one of the contributors to the aging process (Vlassara et al., 2009; Kume et al., 2010). Reduction or dysfunction of Mn-SOD-mediated mitochondrial oxidative stress may be involved in the pathogenesis associated with the aging kidney.

Lim et al. (2012) reported increased oxidative stress in the kidneys of aged (24 months old) mice compared with that observed among younger mice. This finding was associated with a reduction in Cu/Zn-SOD and Mn-SOD expression in the kidney, which may be mediated by decreases in SIRT1, PGC-1α, estrogen-related receptor (ERR)-1α, PPARα, and Klotho. Klotho has been identified as an antiaging gene that is primarily expressed in the kidney, parathyroid, and choroid plexus (Kuro-o et al., 1997); decreased Klotho expression has been correlated with kidney disease. Klotho exerts resistance against oxidative stress, possibly via the activation of FoxO transcription factors, which lead to increased expression of antioxidative enzymes, including Mn-SOD (Yamamoto et al., 2005). Klotho^–/–^ mice exhibit enhanced nitrotyrosine levels and reduced levels of antioxidative enzymes, including Mn-SOD in the kidney (Kimura et al., 2018). Additionally, Hou et al. (2016) demonstrated that the production of endogenous hydrogen sulfide (H_2_S) in the aging kidney is insufficient. Exogenous H_2_S can reduce renal oxidative stress in aging mice via a mechanism that involves enhanced nuclear translocation of Nrf2 and increased expression of antioxidative enzymes, including Mn-SOD (Hou et al., 2016). Moreover, the endogenous advanced glycated end products (AGEs)/receptor for AGEs (RAGE) axis mediates factors associated with the aging process in many tissues and organs, including the kidney. Teissier et al. (2019) showed that older (20 months old) WT mice exhibited renal inflammation and oxidative stress with reduced expression of Mn-SOD compared with 3-month-old counterparts. By contrast, 20-month-old RAGE^–/–^ mice were characterized by lower inflammation and elevated expression levels of Mn-SOD and SIRT1 in the kidney compared with their 20-month-old WT counterparts (Teissier et al., 2019).

By contrast, Gomes et al. (2009) detected increased levels of NADPH oxidase subunits and H_2_O_2_ in aged Wistar–Kyoto rats (52 weeks old) compared with those in young rats, although aged rats show no renal functional impairment or marked elevation in blood pressure. The aged rats did exhibit increased expression of antioxidative enzymes, including Mn-SOD, extracellular Cu/Zn-SOD, and catalase (Gomes et al., 2009), and these findings reveal that increases in antioxidant defenses may be useful for counteracting the damaging impact of oxidative stress.

#### Other Chronic Kidney Disease Animal Models

Albumin overload is a well-known model of renal tubulointerstitial injury caused by inflammation and fibrosis (Eddy, 1989). In the kidney of albumin-overload mice, Mn-SOD expression is reduced, thus promoting mitochondrial oxidative stress and inflammation. This condition can be improved by treatment with an Mn-SOD mimic [Mn (III)tetrakis(4-benzoic acid)porphyrin chloride (MnTBAP)] (Zhuang et al., 2015; Jia et al., 2016).

Unilateral ureteral obstruction (UUO) in a rodent model generates progressive renal fibrosis (Chevalier et al., 2009). Oxidative stress and inflammation are closely associated with the pathogenesis of UUO-induced renal fibrosis (Chevalier et al., 2009). In the kidney of UUO mice, the expression of antioxidative enzymes, including Mn-SOD, is reduced, resulting in mitochondrial oxidative stress, inflammation, and tubular epithelial–mesenchymal transition (EMT; Li et al., 2017; Liu et al., 2017). This condition can be improved by treatment with MnTBAP (Li et al., 2017; Liu et al., 2017).

## Physiological Significance of Manganese Superoxide Dismutase on Kidney Disease in Human Studies

### Manganese Superoxide Dismutase Gene Polymorphisms on Diabetic Kidney Disease

Ala16Val (rs4880C/T) is a functional Mn-SOD SNP polymorphism in exon 2 of *sod2* gene. The substitution of C to T (GCT to GTT), resulting in the conversion from alanine (A) to valine (V), induces a conformational change in the mitochondrial targeting sequence. This results in less efficient transport of Mn-SOD into the mitochondrial matrix and decreases antioxidant activity within the mitochondria (Shimoda-Matsubayashi et al., 1996; Sutton et al., 2003). The relationship of this Mn-SOD gene polymorphism with DKD has been studied widely.

Previous reports revealed an association between the Mn-SOD gene polymorphism and the risk for developing DKD. Möllsten et al. (2007) reported that VV genotype was associated with an increased risk of diabetic nephropathy among Finnish and Swedish patients with type 1 diabetes. Nomiyama et al. (2003) identified the VV genotype with significantly higher frequency in the diabetic patients with nephropathy compared with the AA or VA genotype; however, this was unrelated to the etiology of type 2 diabetes in Japanese patients. By contrast, Houldsworth et al. (2015) demonstrated that the CC (alanine/alanine) genotype in type 1 diabetic patients might protect against diabetic nephropathy. According to another study on Korean type 2 diabetic patients, the alanine-encoding allele was detected at significantly lower frequency among patients with nephropathy who have micro- or macroalbuminuria compared with those patients without nephropathy (Lee et al., 2006). Liu et al. (2009) showed that the AA and VA genotypes were independent factors that protected against the development of nephropathy in Chinese type 2 diabetic patients. Ascencio-Montiel et al. (2013) demonstrated that in type 2 diabetes patients from Mexico, the frequency of the TT (valine/valine) genotype was 6.7% higher in patients with macroalbuminuria than in those in the normo-albuminuria group; the CC (alanine/alanine) genotype was associated with a lower risk of macroalbuminuria than the TT (valine/valine) genotype (Tian et al., 2011). Moreover, in a meta-analysis that included five studies focusing on type 2 diabetic patients, the alanine-encoding allele was associated with a reduced risk of macroalbuminuria (CR 0.65, 95% CI 0.52–0.80) compared with the valine-encoding allele (Tian et al., 2011). Likewise, a meta-analysis that included 17 published articles with both type 1 and 2 diabetic patients revealed a significant association of the alanine-encoding allele with a reduced risk of diabetic microvascular complications and nephropathy in the dominant model (OR 0.788, 95% CI 0.680–0.914 and OR 0.801, 95% CI 0.664–0.967, respectively) (Ascencio-Montiel et al., 2013).

### Membranous Nephropathy

Membranous nephropathy (MN) is a leading cause of nephrotic syndrome in adults. Glomerular damage is induced by subepithelial immune deposits that consist mainly of IgG_4_ and complement components C5b-9. Several podocyte proteins, including aldose reductase (AR) and Mn-SOD, have been identified as renal antigens associated with the pathogenesis of MN (Prunotto et al., 2010). Confocal and immunoelectron microscopy (IEM) displayed co-localization of anti-AR and anti-Mn-SOD IgG_4_ and C5b-9 in electron-immune deposits in podocytes. Additionally, Murtas et al. (2012) demonstrated that the levels of serum IgG_4_ directed against AR and Mn-SOD in patients with MN are higher in MN than those in any of the other proteinuria-associated glomerulonephritides, including focal glomerulonephropathy and IgA nephritis.

## Mitochondria-Targeted Therapies for Scavenging O_2_^•–^

Mitochondria are both sources and targets of ROS. The dysfunction of mitochondria due to oxidative stress may be the causal factor for kidney disease. As such, it will be necessary to develop pharmacological methods aimed at reducing oxidative damage to mitochondria and their components, including Mn-SOD. Several molecules that accumulate within the mitochondria and can scavenge ROS have been developed to address this issue. The beneficial effect of mitochondria-targeted drugs for kidney injury has been shown in several animal models (Table 2). However, those drugs are not delivered to only the kidney; therefore, the risk of adverse effects in systemic administration remains. To address this issue, it is necessary to develop the drugs that specifically deliver to the kidney.

**TABLE 2:** Mitochondria-targeted drugs and animal models of kidney disease.

Mitochondria-targeted drugs	Antioxidative moiety	Animal models of kidney disease showing the benefit of mitochondria-targeted drugs	References
MitoQ	Ubiquinone	I/R-induced AKI mice	Dare et al., 2015
		Sepsis-associated AKI rats	Lowes et al., 2008
		Cisplatin-induced AKI mice	Mukhopadhyay et al., 2012
		Ins2(+/)^–^(AkitaJ) mice	Chacko et al., 2010
		db/db mice	Xiao et al., 2017; Han et al., 2018
		ADPKD model mice (Ksp-Cre PKD1^flox/flox^) and rats (Han:SPRD Cy/+)	Ishimoto et al., 2017
SkQ1	Plastoquinone	mtDNA mutator mice	Shabalina et al., 2017
SkQR1		I/R-induced AKI rats	Plotnikov et al., 2011
		Gentamicin-induced AKI rats	Jankauskas et al., 2012
SS-31	Tyr or Dmt (2′,6′-dimethyltyrosine) residues	I/R injury rats	Szeto et al., 2011
		PTRA-induced I/R pig	Eirin et al., 2012
MnTMPyP	Manganese metalloporphyrin	I/R injury of rats	Liang et al., 2009
MnTnHex-2-PyP^5+^		I/R-induced AKI rats	Dorai et al., 2011
MnTBAP		Albumin-overload model mice	Zhuang et al., 2015; Jia et al., 2016
		UUO model mice	Liu et al., 2017
Mito-CP	Nitroxide CP	Cisplatin-induced AKI mice	Mukhopadhyay et al., 2012

*MnTMPyP, MnP manganese (III) tetrakis (1-methyl-4-pyridyl) porphyrin; MnTnHex-2-PyP^5+^, Mn (III) meso-tetrakis (N-n-hexylpyridinium-2-yl) porphyrin; MnTBAP, Mn (III) tetrakis (4-benzoic acid) porphyrin chloride; CP, carboxy-proxyl; I/R, ischemic/reperfusion; AKI, acute kidney disease; ADPKD, autosomal dominant polycystic kidney disease; PTRA, percutaneous transluminal renal angioplasty; UUO, unilateral ureteral obstruction.*

### MitoQ

MitoQ is a derivative of ubiquinone conjugated to triphenylphosphonium (TTP), a lipophilic cation that enables this molecule to enter and accumulate within the mitochondria via an electrochemical gradient (Smith et al., 2003; Smith and Murphy, 2010). In the mitochondrial matrix, MitoQ is reduced to the active antioxidant form, ubiquinol, by the respiratory chain, which serves to protect the mitochondria and their components from oxidative damage (Smith et al., 2003; Smith and Murphy, 2010). Additionally, MitoQ is cleared rapidly from the plasma after intravenous administration and accumulates within the kidney (Porteous et al., 2010; Smith and Murphy, 2010). Previous animal studies have revealed the beneficial effects of MitoQ in association with various kidney disorders, including I/R-induced AKI (Dare et al., 2015; Kezic et al., 2016), sepsis-induced AKI (Lowes et al., 2008), cisplatin-induced kidney injury (Mukhopadhyay et al., 2012), DKD (Chacko et al., 2010; Xiao et al., 2017; Han et al., 2018), and polycystic kidney disease (Ishimoto et al., 2017).

### SkQ1 and SkQR1

SkQ1 is a compound of plastoquinonyl-decyl-triphenylphosphonium in which ubiquinone was replaced by plastoquinone (Antonenko et al., 2008). SkQR1 is also a mitochondria-targeted compound and a conjugate of a positively charged rhodamine molecule with plastoquinone (Antonenko et al., 2008). SkQs, including SkQ1 and SkQR1, penetrate planar phospholipid membranes, and as such, they can accumulate in mitochondria (Antonenko et al., 2008). Administration of SkQ1 to mitochondrial DNA mutator mice resulted in diminished renal oxidative stress and mitochondrial dysfunction (Shabalina et al., 2017). In experimental rats with I/R injury, SkQR1 treatment normalized the ROS levels in kidney mitochondria, decreased blood urea nitrogen (BUN) and creatinine levels, and reduced disease-associated mortality, compared with what was observed among the rats without treatment (Plotnikov et al., 2011). Additionally, SkQR1 was also effective for the treatment of gentamicin-induced AKI (Jankauskas et al., 2012).

### SS-31

The Szeto–Schiller (SS)-31 peptide, also known as Bendavia, may optimize the mitochondrial phospholipid cardiolipin microdomains. This may lead to reduced electron leak from the inner membrane (Brown et al., 2013). In an experimental model of I/R injury in the kidney, treatment with SS-31 attenuated renal oxidative stress prevented tubular cell apoptosis and necrosis, and reduced inflammation (Szeto et al., 2011). Rapid ATP recovery in mitochondria secondary to administration of SS-31 led to the protection of microvascular endothelial cells, reduced microvascular congestion, and, thus, better reflow to the medulla. In an experimental pig model featuring percutaneous transluminal renal angioplasty, administration of SS-31 resulted in decreased microvascular rarefaction, apoptosis, oxidative stress, tubular injury, and fibrosis, which were all associated with preserved mitochondrial biogenesis in the kidney after reperfusion (Eirin et al., 2012).

### Manganese Superoxide Dismutase Mimics

Mn-SOD mimics are stoichiometric scavengers of O_2_^•–^ and accumulate in mitochondria depending on their positive charge and lipophilicity (Miriyala et al., 2012). Among the cationic metalloporphyrins, the Mn porphyrins (MnP) are the most potent Mn-SOD mimics; these molecules have been designed and optimized for accumulation in the mitochondria where they mimic the action of the Mn-SOD catalytic site (Miriyala et al., 2012). The MnP manganese (III) tetrakis (1-methyl-4-pyridyl) porphyrin (MnTMPyP) functions as a peroxynitrite scavenger. Administration of MnTMPyP resulted in diminished lipid peroxidation and reduced nitrotyrosine content in the proximal tubular area in association with reductions in caspase-3 activation and tubular epithelial cell damage after I/R injury in rats (Liang et al., 2009). Similarly, administration of Mn (III) meso-tetrakis (N-n-hexylpyridinium-2-yl) porphyrin (MnTnHex-2-PyP^5+^) or Mn (III) tetrakis (4-benzoic acid) porphyrin chloride (MnTBAP) also resulted in protection against the deleterious effects associated with I/R-induced AKI (Dorai et al., 2011) as well as in the albumin-overload kidney injury (Zhuang et al., 2015; Jia et al., 2016) and the UUO model (Liu et al., 2017). Moreover, mitochondria-targeted carboxy-proxyl (Mito-CP) is a five-membered nitroxide CP conjugated to the TTP cation. Administration of Mito-CP resulted in improvements in parameters associated with cisplatin-induced AKI, including kidney dysfunction, renal inflammation, and tubular cell apoptosis, which were equivalent to those of MitoQ (Mukhopadhyay et al., 2012).

## Conclusion and Perspectives

Mitochondrial oxidative stress is induced by an imbalance between ROS production and scavenging; the scavenging function may be impaired or destroyed due to dysfunction of antioxidative defense in mitochondria. Mitochondrial oxidative stress contributes to the pathogenesis of kidney disease, including AKI, CKD, and the AKI to CKD transition, and mitochondria-targeted drugs may suppress the onset or progression of kidney disease (Figure 3). Mn-SOD-associated dysfunction is one of the main factors underlying defective antioxidative defense in mitochondria. Mn-SOD dysfunction results in a net increase in ROS and generates a vicious cycle in which mitochondrial oxidative stress is amplified, as Mn-SOD then becomes a target of dysfunctional oxidative modification. An agent with the capacity to scavenge excess ROS in mitochondria would lead to a break in this vicious cycle, serving to preserve Mn-SOD and mitochondrial function and consequently suppress kidney disease. Although Mn-SOD may become dysfunctional in kidney disease, it remains unclear whether mitochondrial oxidative stress due to only Mn-SOD dysfunction is the primary contributor to the pathogenesis of kidney disease. Redox state in mitochondria is regulated by not only Mn-SOD but also other antioxidative enzymes, including Cu/Zn-SOD, GPx, and the PRx/TRx system. However, as Mn-SOD catalyzes the first step in ROS scavenging in mitochondria, treatments targeted at supporting Mn-SOD integrity and function may lead to effective treatments to prevent the onset and progression of kidney disease.

## Author Contributions

MK designed, wrote, and edited the manuscript. JX, YO, IM, and DK contributed to the discussion. MK was the guarantor of this work. All authors contributed to the article and approved the submitted version.

## Conflict of Interest

Boehringer Ingelheim, Mitsubishi Tanabe Pharma, Kyowa Kirin, Taisho Pharmaceutical Co., and Ono Pharmaceutical Co., contributed to establishing the Division of Anticipatory Molecular Food Science and Technology. The authors declare that the research was conducted in the absence of any commercial or financial relationships that could be construed as a potential conflict of interest.
